# Assessing the Quality of Goal Setting in Behavioural Support for Smoking Cessation and its Association with Outcomes

**DOI:** 10.1007/s12160-015-9755-7

**Published:** 2015-11-24

**Authors:** Fabiana Lorencatto, Robert West, Carla Bruguera, Leonie S. Brose, Susan Michie

**Affiliations:** School of Health Sciences, City University London, London, EC1V 0HB UK; Department of Clinical, Educational and Health Psychology, University College London, London, WC1E 6BT UK; Department of Epidemiology & Public Health, University College London, London, WC1E 6BT UK; National Addictions Centre, UK Centre for Tobacco and Alcohol Studies, Institute of Psychiatry, King’s College London, London, SE5 8BB UK

**Keywords:** Goal setting, Smoking cessation, Quality, Fidelity, Behavioural support

## Abstract

**Background:**

Smoking cessation behavioural support can be effective but practitioners differ markedly in effectiveness, possibly due to variation in the quality of delivery of key behaviour change techniques, such as goal setting (i.e. setting a quit date).

**Objectives:**

This study aimed to (i) develop a reliable method for assessing the quality of practitioners’ support in setting quit dates and (ii) assess whether quality predicts initiation of abstinence as a first step to quitting.

**Methods:**

A scale for scoring the quality of goal setting was developed from national guidance documents and applied to 85 transcribed behavioural support sessions. Inter-rater reliability was assessed. Associations between quality scores and quit attempts were assessed.

**Results:**

The 10-item scale produced had good inter-rater reliability (Kappa = 0.68). Higher quality goal setting was associated with increased self-reported quit attempts (*p* < .001; OR = 2.60, 95 % CI 1.54–4.40). The scale components ‘set a clear quit date’ (*χ*^2^ (2, *N* = 85) = 22.3, *p* < .001) and ‘within an appropriate timeframe’ (*χ*^2^ (2, *N* = 85) = 15.5, *p* < .001) were independently associated with quit attempts.

**Conclusions:**

It is possible to reliably assess the quality of goal setting in smoking cessation behavioural support. Higher quality of goal setting is associated with greater likelihood of initiating quit attempts.

## Introduction

Behaviour change interventions are complex, featuring multiple, potentially interacting, component behaviour change techniques. [1, 2] The implementation of such complex interventions on a large scale or in clinical practice is rarely consistent or straightforward. [3, 4] It has been demonstrated that when interventions are consistently delivered as intended, they will produce better results than when delivery is poor or variable [5–7]. Comprehensively assessing the implementation of an intervention requires assessing both whether or not active components of behaviour change interventions are delivered (i.e. *fidelity* of delivery), and also how well interventions are delivered (i.e. *quality* of delivery) [6, 8, 9]. A number of conceptual models of intervention fidelity have been proposed, which provide standardised definitions alongside detailed recommendations for assessing fidelity [8]. However, there is a lack of standard definitions or methods for assessing the quality with which behaviour change interventions are delivered.

In the psychotherapy literature, there are numerous examples of strategies for assessing the quality of delivery of interventions such as cognitive behavioural therapy [10, 11]. These strategies typically equate the quality of intervention delivery with the notion of intervention provider *competence* (i.e. the knowledge/skills for adequately delivering intervention components) [11], and often take the form of two-staged ‘clinical practice assessments,’ whereby the requisite competences for optimally delivering an intervention are first specified and then observed in actual or simulated clinical practice. The quality of delivery is subsequently rated using standardised competence assessment scales, such as the ‘cognitive therapy adherence and competence scale’ [10–13]. Adopting a similar approach, this study aims to develop a method for examining the quality with which smoking cessation behavioural support is delivered in clinical practice.

Behavioural support for smoking cessation has been shown to be a highly cost-effective, life-preserving intervention when delivered across various modalities [14–18]. Behavioural support consists of advice, discussion and activities aimed at minimising a smoker’s motivation to smoke, maximising the resolve not to smoke, helping with strategies to minimise exposures to smoking cues, coping with urges when they occur, and making the best use of adjuvant activities such as smoking cessation medications [19]. Behavioural support is widely implemented in clinical practice in the UK, via a network of 152 National Health Service (NHS) Stop Smoking Services and telephone quitline services offering medication and weekly one-to-one or group behavioural support sessions [20]. However, there is a substantial variation in outcomes across NHS Stop Smoking Services (i.e. 3–66 % successful quit rates) [21], and evidence that smoking cessation behavioural support is often not delivered consistently or in accordance with recommended practice (i.e. with low fidelity). For example, the analysis of transcripts of audio-recorded NHS Stop Smoking Service and quitline sessions with trained stop-smoking practitioners show that, on average, approximately 50 % of behaviour change techniques specified in the treatment manuals are delivered [22, 23].

Although this evidence suggests that fidelity of delivery is poor, the quality with which behaviour change techniques are delivered for smoking cessation behavioural support interventions is unclear. Evidence-based competences for delivering behavioural support interventions have been identified for both individual- and group-based support [24, 25]. These provide standards against which the quality of delivery of smoking cessation interventions may be evaluated [11]. However, there have been few studies assessing the extent to which these competences are demonstrated when delivering behavioural support interventions. One of the first attempts to systematically assess the quality of delivery of behavioural support for smoking cessation used a six-point scale to rate transcripts of audio-recorded sessions in terms of delivery of behaviour change techniques (i.e. fidelity) and the competence with which such techniques were delivered (i.e. quality) [26]. The scale was piloted on 36 session transcripts in the context of a process evaluation for a research trial. There is a need to establish similar methods for monitoring quality in the context of routine clinical practice and to assess the reliability of the method.

The current study aimed to develop a reliable method for assessing the quality of delivery in clinical practice of a key behaviour change techniques for smoking cessation behavioural support, ‘goal setting’. Goal setting in smoking cessation typically involves setting a quit-date with the smoker, which is the date on which the smoker will initiate their quit attempt by engaging in complete abstinence from that point onwards [19]. There is both a theoretical and empirical rationale for initially focusing on goal setting as a key behaviour change technique. PRIME theory [27] argues that self-regulation is integral to successfully quitting in that ex-smokers have to continuously maintain their resolve not to smoke, whilst coping with urges and withdrawal symptoms in order to prevent relapse. Control theory [28] posits that setting goals and revising goals in the light of feedback are key components of a self-regulation loop. Goal setting has also been identified as a key evidence-based competence for delivering smoking cessation behavioural support. [24]

Furthermore, the extent to which ‘better’ goal setting as delivered is associated with improved outcomes for smoking cessation behavioural support is unknown. In other domains, goal setting has been associated with improved intervention outcomes. For example, the enactment of goal setting by intervention recipients as part of a type 2 diabetes intervention has been associated with a significant reduction (≥5 %) in body mass index. [29] Thus, a secondary aim of this study was to examine the association between the quality of goal setting and the likelihood of smokers initiating a quit attempt as planned, as a first step towards quitting. The lack of goal clarity or setting a quit date too far ahead in the future may be expected to militate against initiation of a quit attempt. The study was conducted in the context of behavioural support delivered by a UK telephone quitline service.

## Methods

### Stage 1: Development and Piloting of the Quality of Goal Setting Rating Scale

#### Sample and Materials

Four national guidance documents were used as a basis for specifying the components of appropriate goal setting. Three were from the UK National Centre for Smoking Cessation and Training (NCSCT; see www.ncsct.co.uk): the ‘Standard Treatment Programme’, the NCSCT’s ‘Training Standard: Learning Outcomes for Training Stop Smoking Practitioners’ and the curriculum of the NCSCT’s knowledge and skills training and accreditation programme [30, 31]. The recommendations about the format and content of behavioural support outlined in these documents are based on evidence-based smoking cessation competences [24, 25]. The fourth document was the treatment manual of a UK national telephone quitline service, which specifies the content, format and procedures that their practitioners are expected to adhere to when delivering behavioural support to smokers.

Audio recordings of behavioural support sessions were obtained from a national quitline service. Sessions were delivered by six practitioners, five of whom had passed the NCSCT’s accreditation programme. Consecutively delivered pre-quit sessions of 110 smokers were audio recorded over 8 months. This minimised the risk of practitioners selecting particular smokers or sessions to record. Informed consent was obtained from both practitioners and smokers prior to recording the session. Only pre-quit sessions were examined, as this is when smokers typically agree to set a quit date, which is usually set for 1 to 2 weeks after this. Eleven of the records were excluded as they were incomplete. A further 14 recordings were excluded for smokers who explicitly stated that they did not wish to set a quit date, resulting in a final sample of 85 recordings that were transcribed verbatim and anonymised.

Practitioners also collected data on the smokers’ general demographic and smoker characteristics using the national quitline service’s standard intake/assessment form for new clients, which is routinely used by practitioners in the pre-quit session. This included smokers’ self-reported time spent with urges (Likert-type scale, 1—none of time to 5—almost all of time), strength of urges (1—no urges to 5—extremely strong urges), commitment to quit attempt (1—low to 4—very high), confidence in quitting (1—low to 4—very high), weeks since most recent quit attempt and length of most recent quit attempt (weeks) (Table 2).

#### Procedure

Two researchers (FL, CB) used a reliable taxonomy of behaviour change techniques [32] to independently identify and characterise the components of comprehensive and appropriate (i.e. quality) goal setting as specified in the three guidance documents and service treatment manual. Those components identified by both researchers across all four documents formed the basis for the quality of goal setting rating scale.

This scale was piloted on 85 transcripts of pre-quit sessions. Two researchers (FL, CB) identified the relevant segment of each transcript in which the practitioner and smoker discussed setting a quit date. All excerpts were then independently scored by both researchers using the developed scale to rate the quality with which practitioners facilitated the process of setting a quit date. Reliability was assessed on all 85 transcripts to ensure coding was reliable and the quality score agreed before proceeding to quality and outcome analysis (stage 2).

#### Analyses

Inter-rater reliability was assessed using weighted Cohen’s kappa [33]. Disagreements were resolved through discussion or consultation with a behaviour change expert (SM).

### Stage 2: Investigating the Association Between the Quality of Goal Setting and Outcomes

#### Sample and Materials

The same set of 85 transcripts scored for quality using the developed rating scale was used to examine the association between the quality of goal setting and initiation of quit attempts.

#### Procedure

Anonymised data were obtained from the quitline as to whether or not smokers in the study made their quit attempt on their agreed date; this was assessed by practitioners on the basis of smokers’ self-report at the follow-up quit date session. Information on demographic (i.e. age, gender, occupational grade, ethnicity) and smoker characteristics (i.e. cigarettes per day, time to first cigarette, pharmacological support, strength of and time spent with urges, commitment and confidence in quitting and weeks since and length of most recent quit attempt) were collected for each smoker by the practitioners using a standardised service monitoring form during routine intake assessments.

#### Analyses

Analyses were conducted in MLwiN version 2.14 and SPSS version 21. A two-level logistic regression model was used to examine the extent to which the quality of goal setting predicted smokers’ quit attempts. Multi-level logistic regression analyses were used to account for clustering that may occur because sessions delivered by the same practitioner are likely to share some similarities. Thus, level 1 was the individual smoker’s treatment episode and level 2 the individual practitioner. Smokers lost to follow-up were treated as still smoking; this is standard practice given loss to follow-up is closely associated with resumption of smoking [34].

To identify whether individual components of the quality of goal setting rating scale were independently associated with quit attempts, each scale component was scored as present/absent within each excerpt, and the association between each component and quit-attempts examined separately using chi-square analyses.

To identify potential confounding variables to control for in the multi-level logistic regression analyses, associations between demographic and smoker characteristics with outcome and predictor variables were examined using *t* tests, chi-squares and ANOVAs as appropriate.

## Results

### Stage 1: Development and Piloting of the Quality of Goal Setting Rating Scale

Seven components contributing to appropriate and comprehensive goal setting (i.e. ‘high’ quality) and three components representing activities resulting in inappropriate goal setting (i.e. ‘low’ quality) were identified across the three guidance documents and service treatment manual, producing a quality of goal setting rating scale of 10 components (Table 1). Scoring using this scale is conducted by allocating points for the delivery of each appropriate component and deducting points for delivery of inappropriate components. Potential scores therefore range from −3 (i.e. delivery of solely inappropriate goal setting components) to 7 (comprehensive delivery of all appropriate goal setting components).

**Table 1:** The quality of goal setting rating scale

	Assessing the quality of goal setting for smoking cessation behavioural support Key features: Help the smoker to set a quit date and goals that support the aim of remaining abstinent.
	Components of competent goal setting: The practitioner should prompt the smoker to set a quit date. The practitioner should then work collaboratively with the smoker to agree upon a suitable quit date. The assigned quit date should be a clear date (i.e. dd/mm/yy), linked to a clear time frame within the near future, ideally within 1–2 weeks following the initial pre-quit session, and should allow sufficient time for the smoker to obtain any smoking cessation medications they plan to use during the quit attempt. The practitioner should outline the rationale as to why gradual cessation/cutting down does not work, and encourage the smoker to smoke as normal up until the agreed quit date. It should be clearly emphasised to the smoker that the goal is not to smoke a single cigarette after the quit date, not even a single puff. The practitioner should support these explanations with examples and normative information as to what other smokers’ found helpful when setting a quit date.
	Scoring: Score 0 if goal setting is completely absent in the content of behavioural support delivered by the practitioner. Additional points are to be incrementally allocated for the delivery of components representing appropriate goal setting (+). Points are to be deducted for the delivery of components contributing to inappropriate goal setting (−1). Possible score range −3 to 7.
0	Absence of goal setting
+1	Prompts goal setting (i.e. encourages smoker to set a quit date)
+1	Agreed quit date is a clear date (i.e. dd/mm/yy)
+1	Agreed quit date is within an appropriate time frame (i.e. within 1–2 weeks of pre-quit session)
+1	Practitioner takes into account the time taken to obtain medication when selecting an appropriate quit date.
+1	Provides advice as to why cutting down does not work
+1	Emphasises that the goal is not to smoke a single cigarette after the quit date, not even a single puff
+1	Provides relevant normative information and examples (i.e. what other smokers’ have found helpful when setting a quit date, research findings regarding effectiveness of suggested behavioural strategies and medications).
−1	Inappropriate goal setting [i.e. not a clear quite date (i.e. dd/mm/yy), not within 1–2 weeks of pre-quit session and/or does not allow sufficient time for smoker to obtain medication]
−1	Encourages or reinforcing cutting down

The weighted Cohen’s kappa was 0.68, representing ‘substantial’ agreement between raters when scoring session excerpts using the developed scale [35].

### Stage 2: Investigating Association Between the Quality of Goal Setting and Outcomes

#### Demographic and Smoker Characteristics

Six practitioners delivered the sessions. Practitioner 01 (P01) delivered 25 sessions, P02 11 sessions, P03 24 sessions, P04 7 sessions, P05 15 sessions and P06 3 sessions. The demographic and smoker characteristics of the smokers are presented in Table 2. At follow-up, only 21 % of the 85 smokers reported initiating an agreed quit attempt as planned, with the rest reported to be currently smoking. There were no significant differences in the characteristics of smokers who did and did not initiate a planned quit attempt. No demographic or smoker characteristics were associated with the quality of goal setting.

**Table 2:** Smoker characteristics, presented overall and by outcome

	Overall sample (*n* = 85)	No quit attempt (*n* = 67; 78.8 %)	Quit attempt (*n* = 18, 21.2 %)
Age, M (SD)	44.3 (16.7)	45.2 (16.1)	40.8 (18.9)
Male, % (*n*)	42.4 [36]	41.8 [28]	44.4 [8]
Occupational grade, % (*n*)			
Employed	49.4 [45]	49.3 [33]	55.6 [9]
Unemployed	42.3 [36]	43.2 [29]	38.9 [7]
Student	7.1 [6]	7.5 [5]	5.6 [1]
Unable to code	1.2 [1]	–	5.6 [1]
Ethnicity, % (*n*)^a^			
White British	85.9 (73)	86.4 (57)	88.9 [16]
Any other White background	9.5 [8]	9.1 [6]	11.1 [2]
Indian	3.5 [3]	4.5 [3]	–
Pharmacological support, % (*n*)			
None	21.7 [18]	21.2 [14]	23.5 [4]
Single NRT	20.5 [17]	21.2 [14]	17.6 [3]
Combination NRT	53 [42]	51.5 [34]	58.8 [10]
Champix	4.8 [4]	6.1 [4]	–
Cigarettes per day, M (SD)	17.3 (10.5)	17.0 (9.9)	19 (13.3)
Time to first cigarette, % (*n*)			
60+ min	18.3 [15]	16.9 [11]	23.5 [4]
31–60 min	14.6 [12]	12.3 [8]	23.5 [4]
6–30 min	40.2 [33]	43.1 [28]	29.4 [5]
<5 min	26.8 [22]	27.7 (65)	23.5 [4]
Time spent with urges, % (*n*)			
None	3.6 [3]	3.0 [2]	5.6 [1]
A little of the time	11.9 [10]	10.6 [7]	16.7 [3]
Some of the time	46.4 [39]	43.9 [29]	50.0 [9]
A lot of the time	28.6 [24]	31.8 [21]	22.2 [4]
Almost all of the time	9.4 [8]	10.6 [7]	5.6 [1]
Strength of urges, % (*n*)			
No urges	4.8 [4]	4.5 [3]	5.6 [1]
Slight	8.3 [7]	6.1 [4]	16.7 [3]
Moderate	35.7 [30]	36.4 [24]	33.3 [6]
Strong	37.6 [32]	39.4 [26]	33.3 [6]
Extremely strong	12.9 [11]	13.6 [9]	11.1 [2]
Commitment to quit attempt, % (*n*)			
Low	3.6 [3]	3.1 [2]	5.9 [1]
Moderate	12.2 [10]	12.3 [8]	11.8 [2]
High	40.2 [33]	40.0 [26]	41.2 [7]
Very high	43.9 [16]	44.6 [29]	41.2 [7]
Confidence in quitting, % (*n*)			
Low	12.5 [10]	12.5 [8]	12.5 [2]
Moderate	36.3 [29]	34.4 [22]	43.8 [7]
High	30 [24]	34.4 [22]	12.5 [2]
Very high	21.3 [17]	18.8 [12]	31.3 [5]
Weeks since most recent quit attempt, M (SD)	92.3 (158.1)	84.3 (144.47)	118.2 (199.9)
Length of most recent quit attempt (weeks), M (SD)	10.1 (16.8)	9.1 (12.7)	14.2 (28.1)

*M* mean, *SD* standard deviation, *NRT* nicotine replacement therapy

^a^Ethnicity is self-reported using the standard UK Census (2001) categories

### Quality of Goal Setting

Across all smokers, the average quality of goal setting score was 1.6 (SD 1.2; range −1 to 5); given the possible range of scores (−3 to 7), this represents ‘low’ quality of goal setting. The average quality score for smokers making a quit attempt was 2.2 (SD .70; range 1 to 4) and 1.4 (SD 1.27; range −1 to 5) in smokers who did not report initiating a quit attempt.

#### Association Between the Quality of Goal Setting and Quit Attempts

Higher quality of goal setting in pre-quit sessions was strongly associated with an increased likelihood of smokers reporting that they made a quit attempt (*p* < .001; OR 2.60, 95 % CI 1.54 to 4.40). The variance partition coefficient indicated no significant differences amongst individual practitioners, with only 2.8 % of variance in quit attempts was explained by the practitioner level. Furthermore, a residual plot demonstrated that there were no significant practitioner differences.

### Association Between the Individual Quality of Goal Setting Rating Scale Components and Quit Attempts

The frequency with which each scale component was identified across excerpts and their association with quit attempts is presented in Table 3. Three of the 10 scale components were independently associated with reported quit attempts. These were ‘*set a clear quit date with the smoker* (i.e. *dd*/*mm*/*yy*)’ (*χ*^2^ (2, *N* = 85) =22.3, *p* < .001), ‘*agree a quit date within an appropriate timeframe* (i.e. *1 to 2 weeks*)’ (*χ*^2^ (2, *N* = 85) =15.5, *p* < .001) and the converse of these ‘*inappropriate goal setting* (i.e. *setting an unclear quit date*, *within an inappropriate time frame or not permissive of sufficient time to obtain medications*)’ (*χ*^2^ (2, *N* = 85) =21.1, *p* < .001) (Table 3).

**Table 3:** Association between individual scale components and quit attempts

Scale component	Frequency (*n* transcripts; max = 85)	Quit attempts made when component delivered; (*n* %)	Quit attempts made when component not delivered; (*n* %)	Pearson chi-square	*p* value
Prompt goal setting	85	19 (22 %)	66 (78 %)	–	–
Set a clear quit date (dd/mm/yy)	40	18 (45 %)	1 (2 %)	22.3	*p* < .001
Set appropriate quit date (time frame within 1–2 weeks of pre-quit session)^c^	52	19 (37 %)	0 (0 %)	15.5	*p* < .001
Considers time taken to obtain medication when setting quit date	30	5 (17 %)	14 (26 %)	0.95	*p* = .331
Advise against cutting down	5	1 (20 %)	18 (22.5 %)	0.02	*p* = .896
Emphasise ‘not a puff’	5	0 (0 %)	19 (23.8 %)	1.52	*p* = .216
Provide normative information	11	2 (18 %)	17 (23.1 %)	0.13	*p* = .722
Inappropriate goal setting (i.e. not clear date, +2 weeks away from pre-quit session)	44	1 (3 %)	18 (43 %).	21.2	*p* < .001
Encourage cutting down	2	0 (0 %)	19 (23.1 %)	0.59	*p* = .443
Undermine commitment to quit attempt (i.e. imply flexibility in quit date)	20	4 (20 %)	15 (23.1 %)	0.08	*p* = .773

## Discussion

A reliable measure of the quality of delivery of a key behaviour change technique in behavioural support for smoking cessation, goal setting, was developed in a national telephone quitline service. A nearly three-fold increase in the likelihood of smokers reporting a quit attempt was observed when goal setting was delivered with higher quality. Specifically, there was a greater likelihood of smokers initiating a planned quit attempt if a specific quit date, within an appropriate time frame, was agreed. The levels of reliability achieved when applying the quality of goal setting rating scale are comparable to those observed for competence assessment methods for interventions in other domains, such as psychotherapy and reducing excessive alcohol use [11, 36].

This study showed that the quitline practitioners were not, in general, appropriately setting quit dates with smokers who expressed an interest and willingness to set a quit date. This echoes the low levels of fidelity observed in previous evaluations of the delivery of behavioural support by UK smoking cessation services and quitlines [23, 24]. It is also consistent with findings from behaviour change interventions in other contexts [6, 11], such as motivational interviewing interventions aiming to increase medication adherence for HIV, which have found that intervention providers often fail to demonstrate competence when delivering intervention components in clinical practice [37].

The nearly three-fold increase in the likelihood of smokers reporting quit attempts with higher quality of delivery of goal setting is consistent with previous studies and systematic reviews demonstrating that better implementation is often associated with better intervention outcomes [5, 6, 11]. For example, a meta-analysis of drug prevention interventions found that interventions with good implementation achieved a mean effect size that was 0.34 greater than those with poor implementation [38]. There is also evidence to suggest that variation in therapist performance when delivering cognitive behavioural therapy is a significant factor in explaining treatment outcomes, particularly for evidence-based therapies implemented in routine clinical settings [11, 13, 39, 40].

Although practitioners delivered the technique ‘goal setting’ to all 85 smokers whose session transcripts were examined (i.e. with 100 % fidelity of delivery), the quality with which practitioners delivered this technique was highly variable and generally low. This suggests that although fidelity is a pre-requisite for quality of intervention delivery, demonstrating high fidelity of delivery does not necessarily guarantee or equate to high quality of intervention delivery or improved intervention outcomes.

The multi-item nature of the quality of goal setting rating scale allowed the investigation of dimensions of quality, showing that the individual components ‘setting a quit date within an appropriate time frame’ and ‘setting a clear quit date’ increased the likelihood of quit attempt enactment, whilst the converse ‘setting an inappropriate quit date’ reduced this likelihood. This analysis demonstrates how a complex behaviour change technique may be deconstructed into subcomponents to identify which specific components contribute to effective outcomes (i.e. the ‘active ingredients’). A review of the goal setting literature for primary care practice highlighted a lack of evidence as to how best to implement goal setting [41]. The present results can help to address this gap and contribute to the advancement of goal setting theory by highlighting which are the core components of higher quality goal setting that may be generalised to theorising and constructing similar goal setting scales in other behavioural domains (e.g. physical activity, healthy eating). These include (1) setting a clear, precise goal; (2) a time sensitive goal; (3) encouraging commitment to the goal and not implying flexibility in the set goal; and (4) providing relevant, supporting information (e.g. the rationale, theory, evidence base for the proposed goal, normative examples as to what similar individuals found helpful in achieving the goal).

The establishment of a reliable method for assessing the quality of goal setting extends to clinical practice methods for examining the quality of delivery of smoking cessation behavioural support interventions which were developed in the context of research trials [26]. It also builds upon methods for assessing the fidelity of delivery of smoking cessation behavioural support in practice [23, 24]. Together, these assessment methods provide a set of reliable tools for examining *how much* alongside *how well* the content of behavioural support is delivered in practice. This methodology can inform the wider issue of monitoring and investigating the implementation of behavioural interventions in clinical practice [6, 8]. For example, setting a clear quit date with a smoker (e.g. Monday, July 29, 2014) as opposed to an unclear quit date (e.g. in 3 weeks’ time) and a date that is within 1 to 2 weeks of the initial pre-quit session is a relatively simple procedure to do in practice. This is unlikely to be time consuming and has the demonstrated potential to make a significant impact on the likelihood of a smoker actually making a quit attempt. Many NHS Stop Smoking Services have treatment manuals and client assessment sheets [42]; therefore, these simple, yet effective, components of the developed quality of goal setting rating scale could potentially be provided to smoking cessation practitioners as a checklist or included as items on client assessment sheets to promote and enhance the quality of goal setting with clients.

The presently developed scale represents one initial approach to assessing how well goal setting is delivered for smoking cessation, in terms of the extent to which goal setting is comprehensively and appropriately delivered. Quality is likely to be a complex, multi-dimensional concept, and it is important to consider other dimensions of quality in addition to comprehensiveness and appropriateness. For example, quality might also encompass the notions of whether the behaviour change technique is delivered at the correct point in the session (i.e. timing), or the extent to which contextual factors such as client readiness are considered by the practitioner and used to tailor the delivery of the technique. Although the present findings indicated that there did not appear to be an individual ‘practitioner effect’ contributing to observed quit attempts, it is possible that more general practitioner characteristics and personal style, such as warmth, tone, empathy, reflective listening and rapport building [43, 44], interact with the content of goal setting delivered to reflect overall quality. The quality of goal setting rating scale developed does not attempt to address additional dimensions of quality, such as practitioner warmth and tone that would be better assessed using video or audio recordings of sessions rather than transcripts of audio-recorded sessions, which do not provide non-verbal data. Nonetheless, the developed scale represents an important step towards assessing quality in this context; further research is needed to examine these additional dimensions of quality.

The developed scale provides a method for assessing the quality of delivery for a single behaviour change technique; similar scales should be developed for other evidence-based behaviour change techniques. [42] Furthermore, the components of the developed scale are based on the evidence base and guidelines specific for smoking cessation. Other components may be of relevance and importance to goal setting in other behavioural domains. Therefore, the extent to which these findings may be generalised to behavioural support interventions delivered in contexts other than smoking cessation and the telephone quitline service examined in this study remains to be determined.

Further limitations of this study include that the outcome data as to whether smokers made a quit attempt relied on self-report, which may have a degree of inaccuracy given the evidence of discrepancies between self-reported and biochemically validated smoking status [45, 46]. Another issue is that since the study was exploratory and the data collected opportunistically, the samples were too small to control for confounding variables in analyses. For example, UK quitline sessions have been found to contain on average 14 behaviour change techniques per session [23]. It is possible that techniques other than goal setting, or combination of techniques, contributed to the likelihood of smokers making a quit attempt, either in their own right, or by supporting the delivery of goal setting.

Some individual components of the quality of goal setting rating scale were identified across the 85 transcripts at a low frequency (e.g. ‘advise against cutting down,’ *n* = 5). This limited the ability to separately examine the association of each scale component with quit attempts, providing insufficient statistical power to detect a potentially significant effect for these individual components. Therefore, these components warrant further investigation and should not be dismissed as unimportant to higher quality goal setting on the basis of the present findings.

Lastly, although all smokers included in the present analysis explicitly stated they were interested in setting a quit date, it is possible that smoker characteristics, such as readiness to quit and motivation, may have influenced the extent to which the practitioners were able to deliver components of goal setting to the smoker, raising questions regarding the causal role of delivery of quality goal-setting.

## Conclusions

It is possible to reliably assess the quality with which stop-smoking practitioners deliver a key evidence-based behaviour change technique in clinical practice and relate this to intervention outcomes. The application of scales such as the quality of goal setting rating scale can contribute to quality improvement efforts in clinical practice. Healthcare systems invest considerable resources into quality improvement initiatives that aim to optimise care delivered and maximise effective outcomes [47]. The granularity of measuring the quality of behaviour change technique delivery demonstrated in this study provides a foundation for generating evidence that can inform targeted future training programmes, continuing professional development and service improvement efforts.
